# Terpenoids from Octocorals of the Genus *Pachyclavularia*

**DOI:** 10.3390/md15120382

**Published:** 2017-12-05

**Authors:** Yu-Chia Chang, Jyh-Horng Sheu, Yang-Chang Wu, Ping-Jyun Sung

**Affiliations:** 1 National Museum of Marine Biology and Aquarium, Pingtung 944, Taiwan; jay0404@gmail.com; 2 Greenhouse Systems Technology Center, Central Region Campus, Industrial Technology Research Institute, Nantou 540, Taiwan; 3 Forcean Marine International Corporation, Kaohsiung 800, Taiwan; 4 Department of Marine Biotechnology and Resources, National Sun Yat-sen University, Kaohsiung 804, Taiwan; 5 Graduate Institute of Natural Products, Kaohsiung Medical University, Kaohsiung 807, Taiwan; 6 Research Center for Natural Products and Drug Development, Kaohsiung Medical University, Kaohsiung 807, Taiwan; 7 Department of Medical Research, Kaohsiung Medical University Hospital, Kaohsiung 807, Taiwan; 8 Graduate Institute of Marine Biology, National Dong Hwa University, Pingtung 944, Taiwan; 9 Chinese Medicine Research and Development Center, China Medical University Hospital, Taichung 404, Taiwan

**Keywords:** *Pachyclavularia*, octocoral, cembrane, briarane, briarellin, secosterol, bioactivity

## Abstract

In this paper, we reviewed natural compounds isolated from octocorals belonging to the genus *Pachyclavularia*, including 20 cembrane-, 39 briarane-, and eight briarellin-related diterpenoids, and one secosterol. The chemical constituents of these 68 secondary metabolites, and their names, structures, and bioactivities, along with studies of their biological activities, are summarized in this review. Based on the literature, many of these compounds possess bioactivities, including anti-inflammation properties, cytotoxicity, and ichthyotoxicity, suggesting that they may have the potential to be developed into biomedical agents for treatment.

## 1. Introduction

**Scheme 1:**
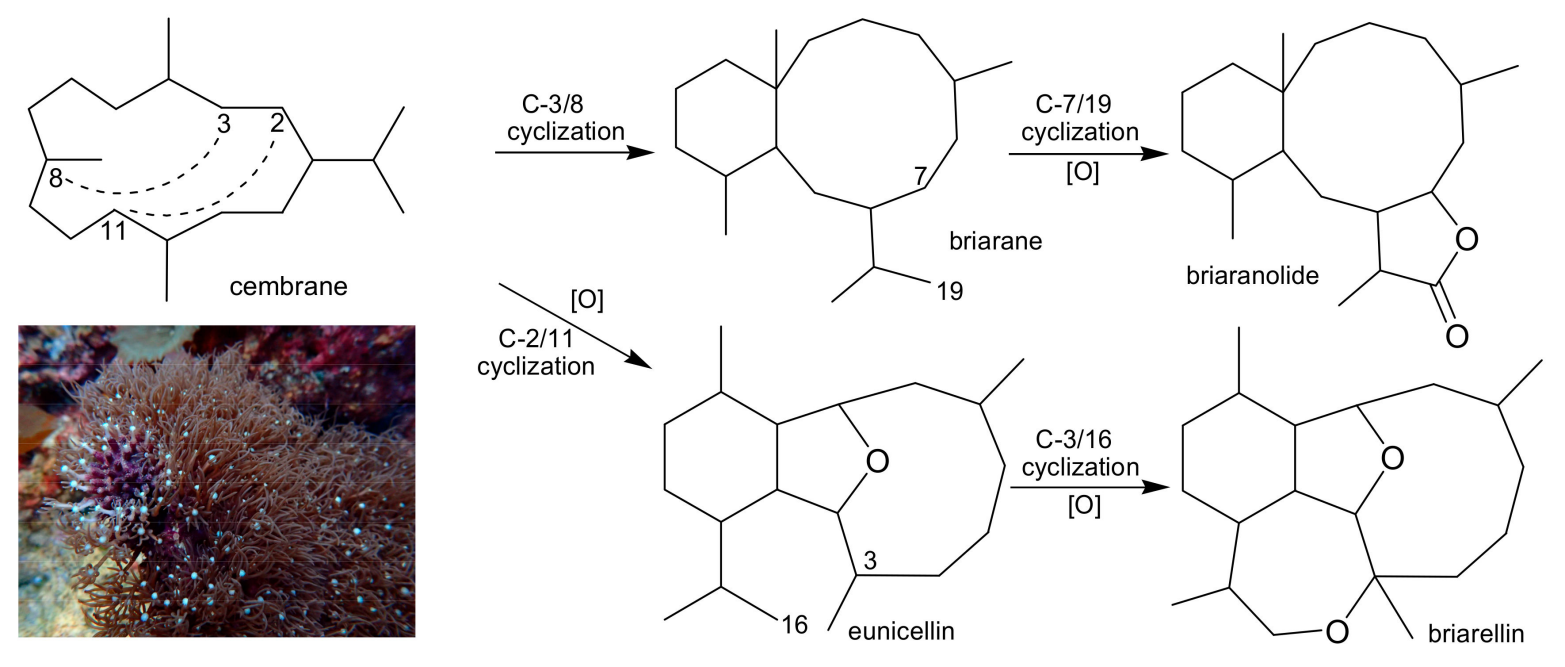
Octocoral *P. violacea* and the proposed biosynthetic pathways of the cembrane, briarane, and briarellin carbon skeletons [4].

Octocorals of the genus *Pachyclavularia* (phylum Cnidaria, class Anthozoa, order Alcyonacea, family Briareidae) [1], which originally belonged to the family Tubiporidae [2], distributed in the subtropical and tropical waters of the Indo-Pacific Ocean, have been investigated. Since the initial study in 1979 discovered a cembranoid diterpene, pachyclavulariadiol (**1**), from the Australian octocoral *P. violacea* [3], subsequent studies over the past few decades have yielded a series of interesting secondary metabolites, particularly diterpenoids originally synthesized from cyclized cembranoids, including briarane- and briarellin-related analogues (Scheme 1) [4]. In this article, we have listed the different types of compounds isolated from two Indo-Pacific octocorals, *Pachyclavularia violacea* (Scheme 1) and *Pachyclavularia* sp., and summarized their chemical constituents and research into the biological activities of these metabolites.

## 2. Cembranoids from *Pachyclavularia violacea* (Quoy and Gaimard, 1833) (=*Clavularia violacea*, *Briareum violaceum*)

**Figure 1:**
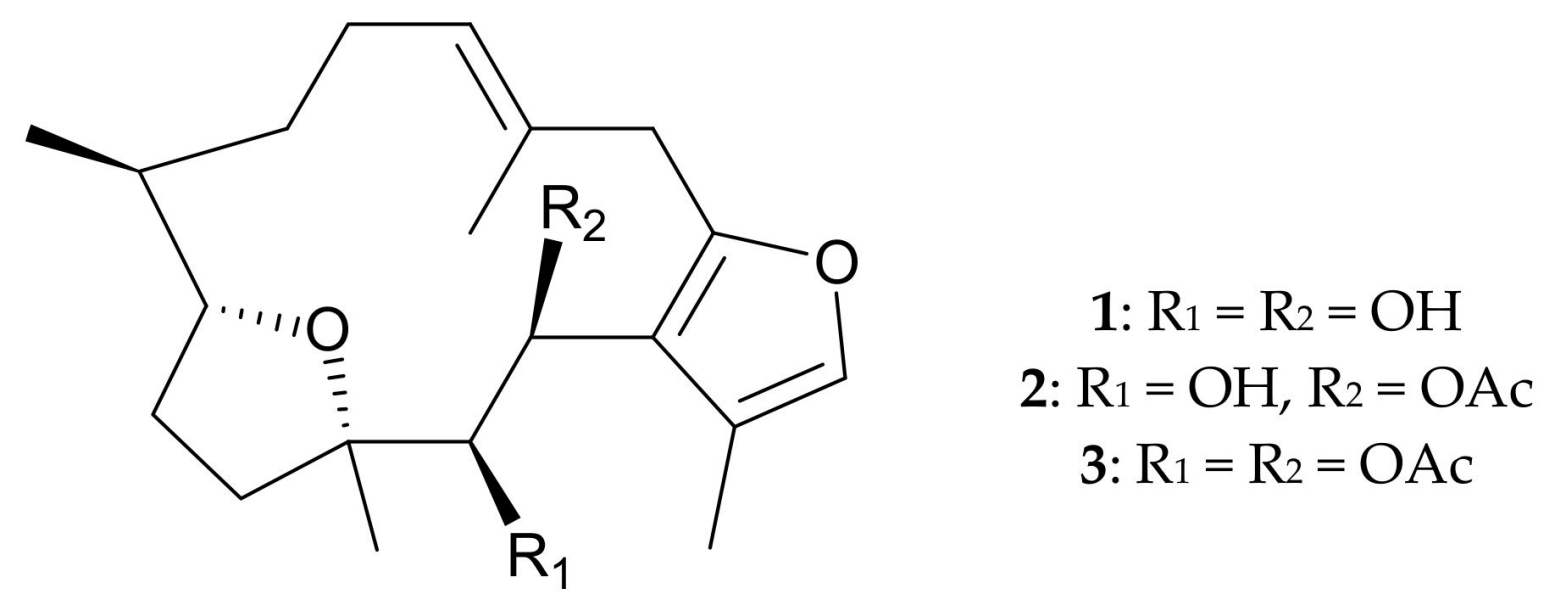
Structures of pachyclavulariadiol (**1**) and its natural mono- and di-acetylated derivatives **2** and **3**.

The *Pachyclavularia* genus includes one very common species, *Pachyclavularia violacea* [2,3]. Bowden and colleagues [3] isolated several compounds, including three new cembrane-type diterpenes, pachyclavulariadiol (**1**), and its naturally-derived mono- and di-acetylated derivatives (**2** and **3**), from octocoral *P. violacea* collected in Australia (Figure 1). The study also established the structures, including the relative configurations, of new furanocembranoids **1**–**3** and determined the configuration of **1** by spectroscopic/chemical methods and X-ray diffraction, respectively.

**Figure 2:**
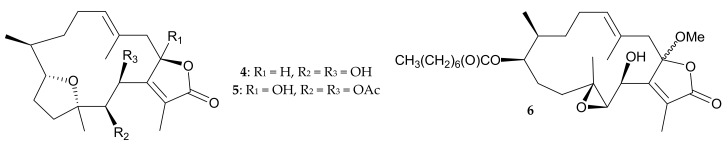
Structures of pachyclavularolide (**4**) and pachyclavulariolides E (**5**) and F (**6**).

In 1989, Inman and Crews [5] reported the isolation of a new cembranolide from *P. violacea* collected from Vanuatu waters, and named the compound pachyclavularolide (**4**) (Figure 2). Later, the structure of **4**, including the relative configuration, was determined using spectroscopic methods and molecular mechanics calculations, and further confirmed by single-crystal X-ray analyses in a study by Sheu et al. [6]. The former study was the first to present a detailed conformational analysis of a 14-membered cembrane ring [5].

Two new cembranes, pachyclavulariolides E (**5**) and F (**6**) (Figure 2), were isolated from *P. violacea* samples collected from waters off Madang in Papua New Guinea in 2000 [7]. The structures of **5** and **6**, including the relative configurations, which were elucidated from spectroscopic data [7], and the structure, including the relative configuration of **5**, was further confirmed from single-crystal X-ray analysis by Sheu et al. [6]. A cytotoxic assay showed that compound **6** had an IC_50_ value of 1.0 μg/mL in the treatment of murine leukemia P388 cells [7], though this compound was claimed to be an artifact due to methanol used in the extraction procedure [7].

**Figure 3:**
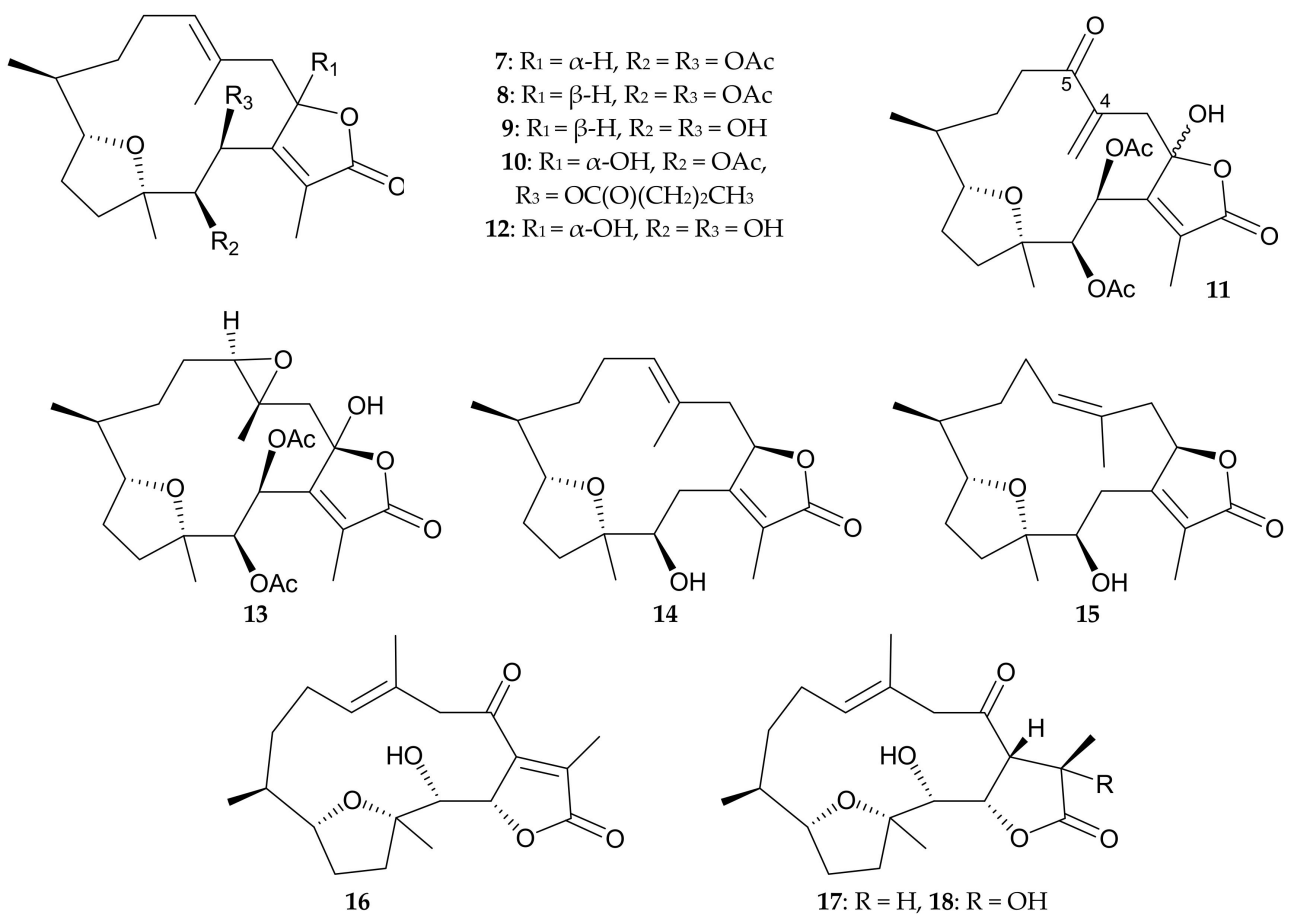
Structures of pachyclavulariolides G–R (**7**–**18**).

Twelve new cembranolides, pachyclavulariolides G–R (**7**–**18**) (Figure 3), and two known analogues, pachyclavulariolide (**4**) and pachyclavulariolide E (**5**) [5,7] (Figure 2), were obtained from *P. violacea* specimens collected from Kenting Coast, Taiwan [6,8]. The structures, including the relative configurations, of cembranolides **7**–**18** were established by spectroscopic/chemical methods [6,8], and the structures of **4**, **5**, **7**, and **8** were also verified by single-crystal analyses [6]. However, the hydroxy group at C-2 in briarane **11** was still unconfirmed at that stage [6], and information regarding the reasonable biosynthetic pathway suggested that briarane **12** could be the precursor of briarane **16** [8]. Cytotoxic assays revealed that pachyclavulariolide K (**11**) had ED_50_ values of 2.8, 3.3, 6.7, and 7.6 μg/mL against P388 (mouse lymphoma), HT-29 (human colorectal adenocarcinoma), A549 (human epithelial carcinoma), and KB cells (human nasopharynx carcinoma), respectvively, while pachyclavulariolides E (**5**), I (**9**), J (**10**), and M (**13**) had ED_50_ values against P388 cells of 4.0, 1.3, 2.5, and 3.2 μg/mL, respectively [6,8].

**Figure 4:**
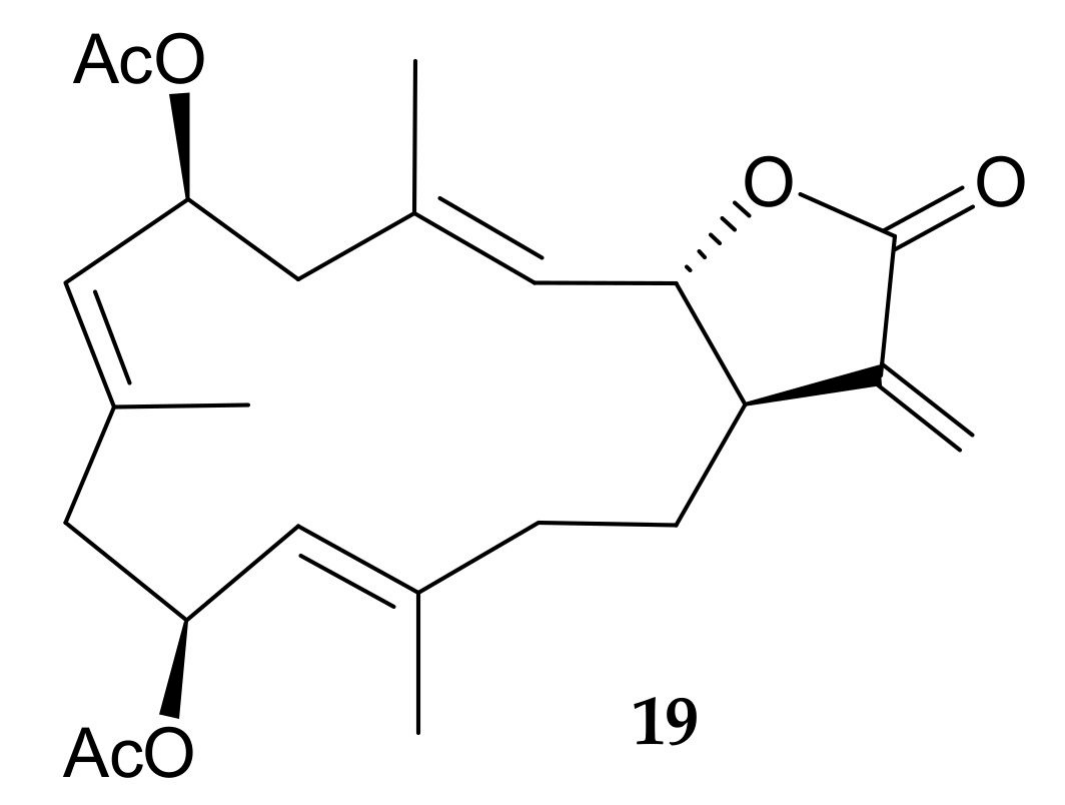
Structure of claviolide (**19**).

A new cytotoxic cembranoid, claviolide (**19**) (Figure 4), was isolated from the dichloromethane extract of *Clavularia violacea*, collected at a depth of 5–6 m off Green Island, Taiwan. The structure, including the relative configuration, of **19** was established using a spectroscopic approach, and this compound was found to exhibit significant cytotoxicity towards P388 and HT-29 tumor cells, with ED_50_ values of 0.84 and 0.38 μg/mL, respectively [9].

**Figure 5:**
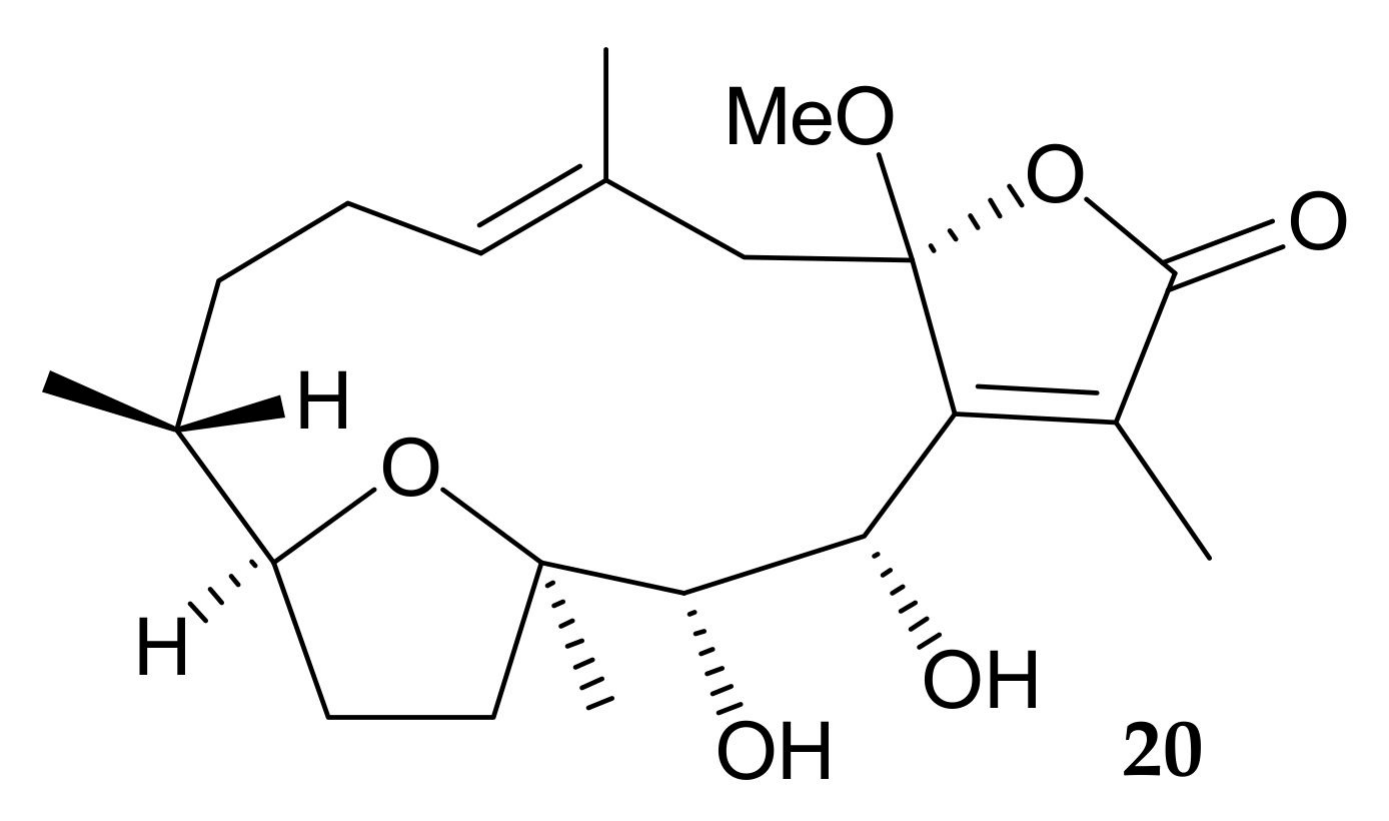
Structure of briaviodiol A (**20**).

Chang and coworkers isolated a new cembranoid, briaviodiol A (**20**) (Figure 5), from the organic extract of *Briareum violaceum* [2] collected off Pingtung Coast, Southern Taiwan, and confirmed its structure, including the relative configuration, by single-crystal X-ray diffraction analysis [10].

Interestingly, with the exception of cembranoids **6** (pachyclavulariolide F) and **19** (claviolide), cembranoids (18/20) from *P. violacea* have been found to possess a tetrahydrofuran moiety, and these furanocembranoids are likely to be chemical markers of cembranoids obtained from *P. violacea*. Among the furanocembranoids, the α,β-unsaturated ketone moiety in pachyclavulariolide K (**11**) seems to enhance cytotoxicity toward various tumor cells.

## 3. Briaranes from *Pachyclavularia* sp. and *Pachyclavularia violacea*

**Figure 6:**
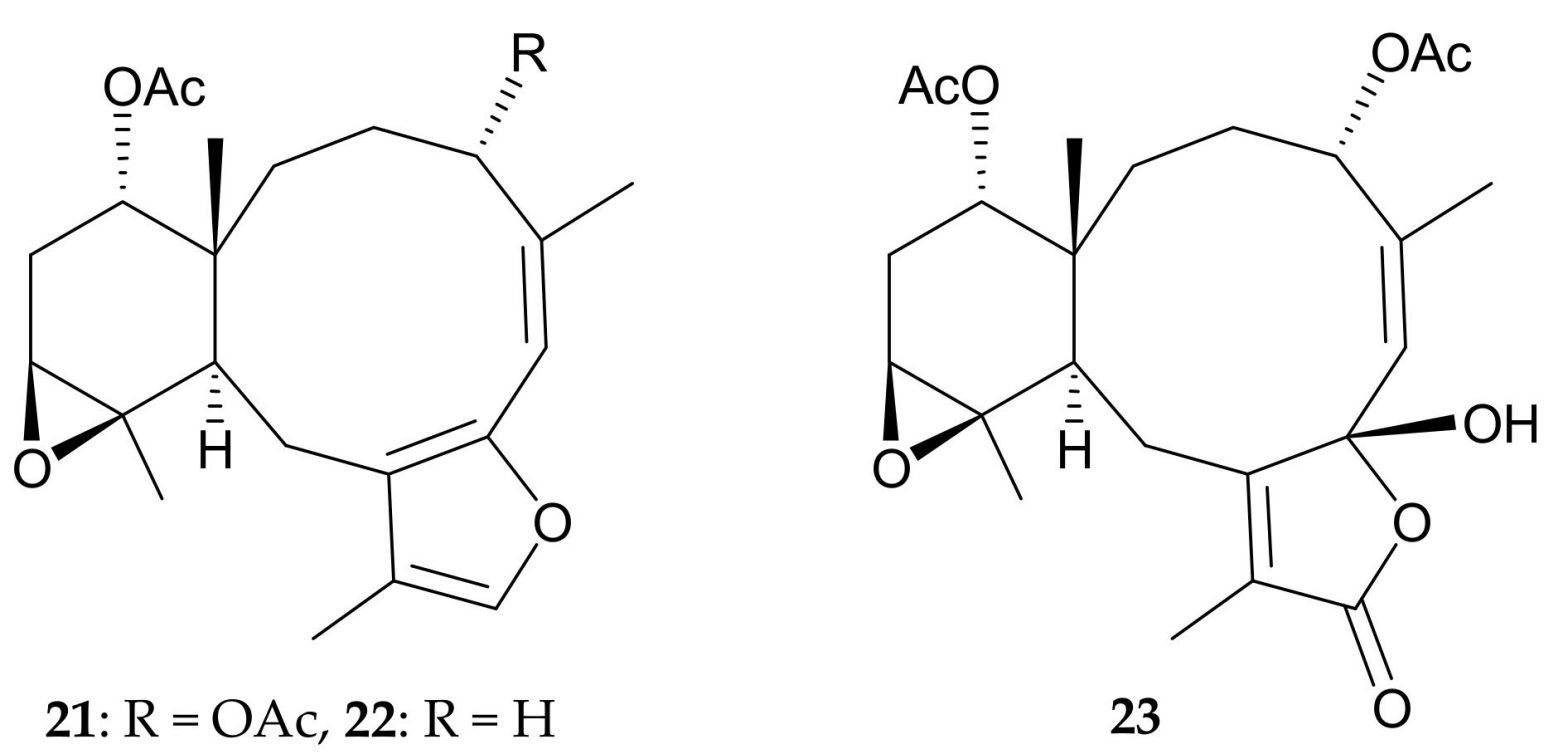
Structures of briaranes **21**–**23**.

Uchio and colleagues [11] identified three new unnamed briaranes, **21**–**23** (Figure 6), from specimens of the octocoral *Pachyclavularia* sp. collected off the Great Barrier Reef, Australia. The authors established the structures of **21**–**23**, including the relative configurations, using a spectroscopic approach, and also found that briarane **21** exhibited ichthyotoxicity towards the mosquito fish *Gambusia affinis*.

**Figure 7:**
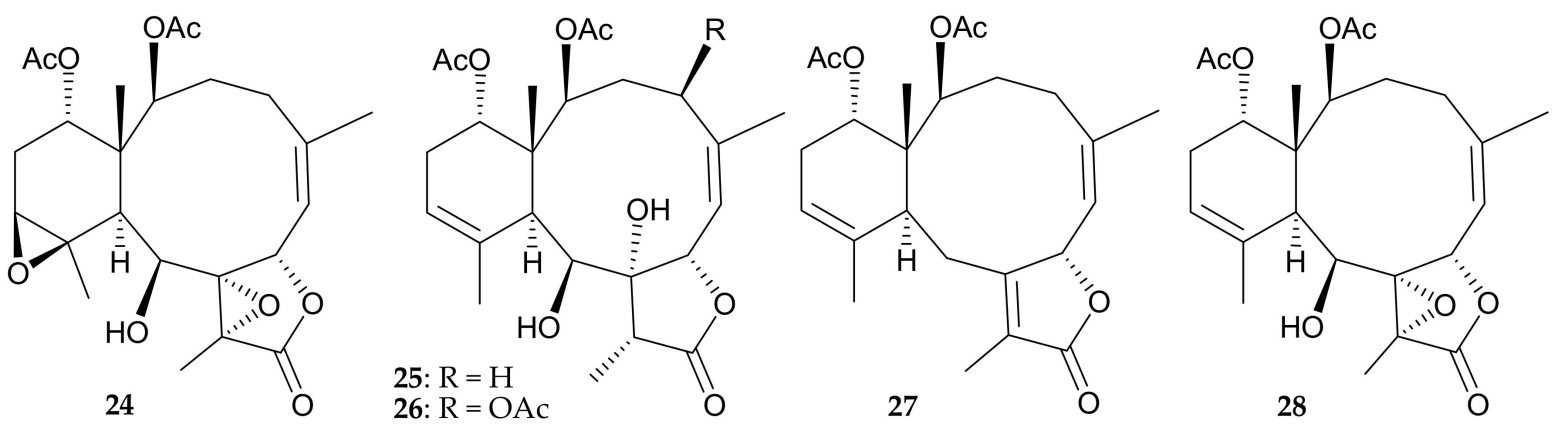
Structures of 2β-acetoxy-2-(debutyryloxy)stecholide E (**24**), 9-deacetylstylatulide lactone (**25**), 4β-acetoxy-9-deacetylstylatulide lactone (**26**), brianthein W (**27**), and 9-deacetylbriareolide H (**28**).

Five briarane diterpenoids, including three new metabolites 2β-acetoxy-2-(debutyryloxy)-stecholide E (**24**), 9-deacetylstylatulide lactone (**25**), and 4β-acetoxy-9-deacetylstylatulide lactone (**26**), along with two known metabolites, brianthein W (**27**) [12] and 9-deacetylbriareolide H (**28**) [13,14] (Figure 7), were isolated from a Taiwanese soft coral *Briareum* sp., which was originally identified as *Pachyclavularia violacea* collected off the coast of Kenting, Taiwan [15]. The structures of new briaranes **24**–**26**, including the relative configurations, were established by chemical/spectroscopic methods. Cytotoxic assays were performed on briaranes **24**–**28** to assess their toxicity against P388, HT-29, KB, and A549 tumor cells, and the results showed that all briaranes except **26** exhibited cytotoxicity against P388 cells, with ED_50_ values of 0.61, 1.12, 0.76 and 0.28 μg/mL, respectively [15], indicating that the acetoxy group at C-4 in **26** redeuce the cytotoxicity. Briaranes **25** and **28** showed cytotoxicity towards HT-29 and KB cells, with ED_50_ values of 1.79 and 0.27 μg/mL, respectively [15]. The cytotoxicities of 9-hydroxybriaranes **24**, **25**, and **28** were not significantly different to their corresponding 9-acetyl derivatives, 2β-acetoxy-2-(debutyryloxy)-stecholide E acetate, stylatulide lactone [16], and briareolide H [13], respectively [15].

**Figure 8:**
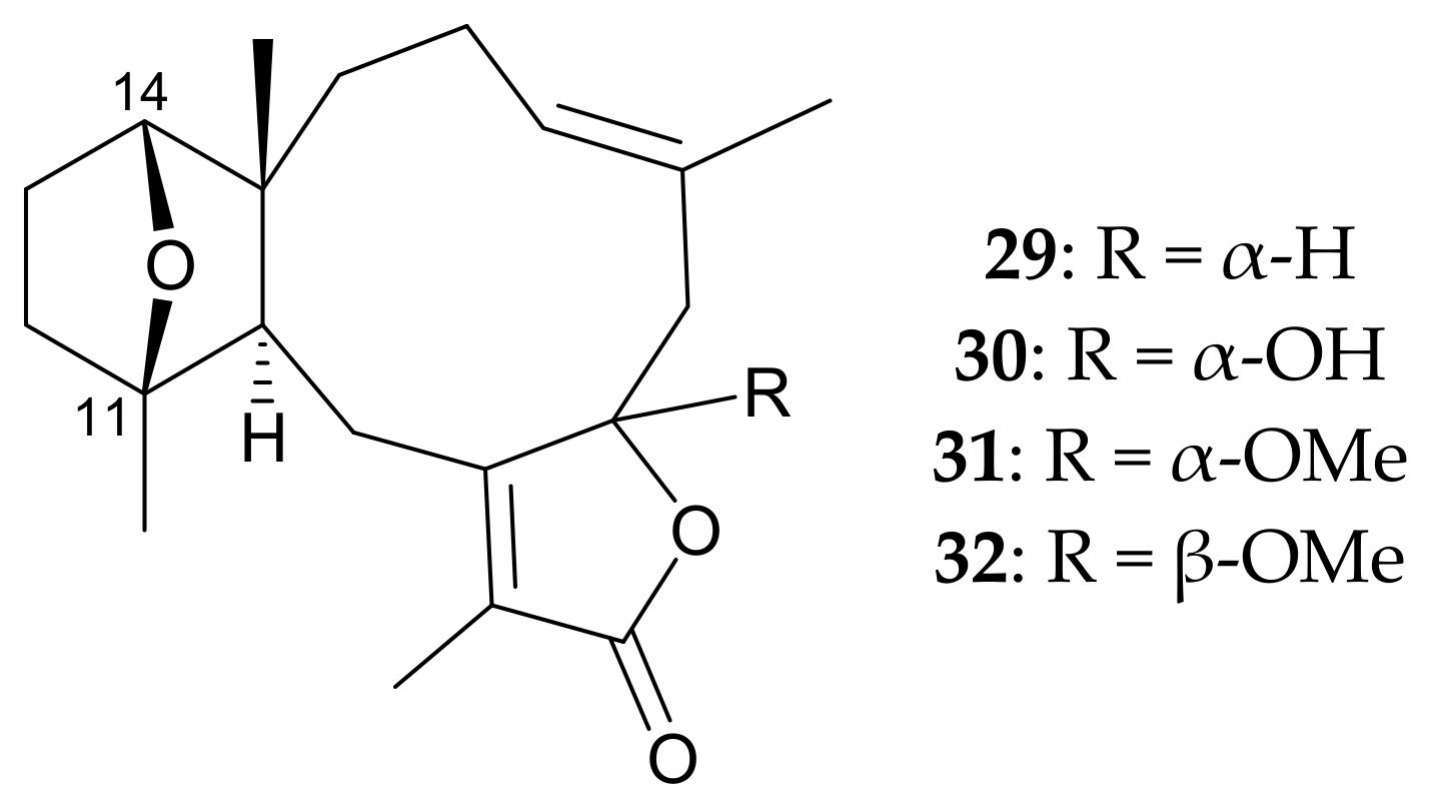
Structures of pachyclavulariolides A–D (**29**–**32**).

In 2000, Xu et al. [7] isolated four new briarane derivatives, pachyclavulariolides A–D (**29**–**32**) (Figure 8), from *P. violacea* specimens collected from reefs off Madang, Papua New Guinea. The authors used a spectroscopic approach to determine the structures, including the relative configurations, of **29**–**32**, and further employed single-crystal X-ray diffraction analysis to identify the structure, including the relative configuration, of briarane **30** (pachyclavulariolide B). They also found that briaranes **29**–**32** were the first briaranes of this type to possess an 11,14-ether bridge that resulted in an oxanorbornane structure.

**Figure 9:**
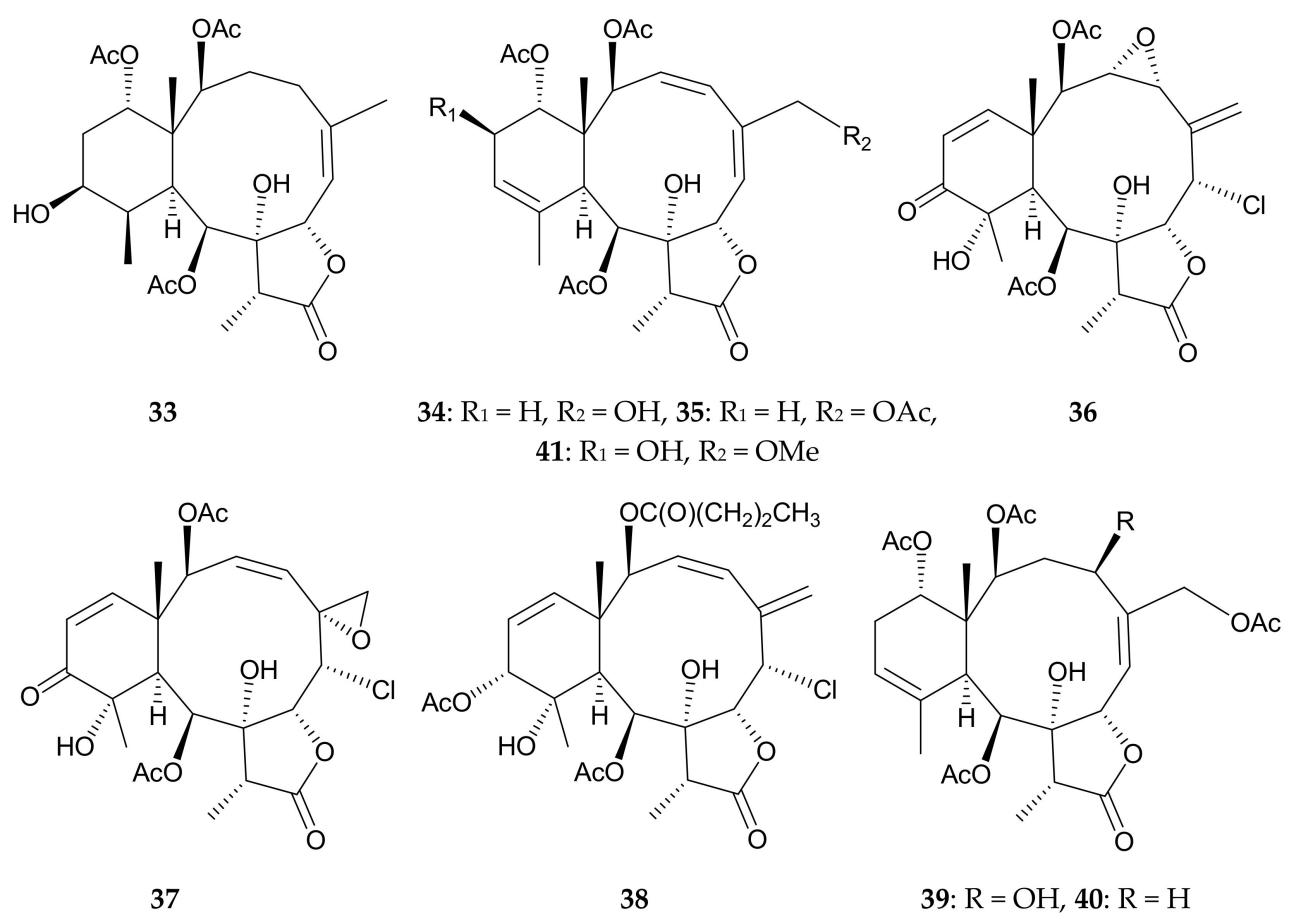
Structures of pachyclavulides A–I (**33**–**41**).

Research by a group in Japan identified nine new briaranes, pachyclavulides A–I (**33**–**41**) (Figure 9), from *P. violacea* collected off Okinawa, and determined their structures, including the relative configurations, using spectroscopic methods [17,18]. The absolute configuration of pachyclavulide A (**33**) was determined by single-crystal X-ray diffraction analysis of its *p*-bromobenzoyl ester [17]. Pachyclavulides B (**34**) and E (**37**) were found to show cytotoxicity against human central neural system (CNS) SNB-75 and A549 (human alveolar epithelial carcinoma) tumor cells, with GI_50_ values of 5.2 and 5.1 μM, respectively [18] and pachyclavulide C (**35**) was not cytotoxic toward SNB-75 cells. It seems that the hydroxy group at C-16 in briarane **34** will enhance the cytotoxity. Enantioselective preparation of the six-membered ring of pachyclavulide B (**34**) was also achieved [19].

**Figure 10:**
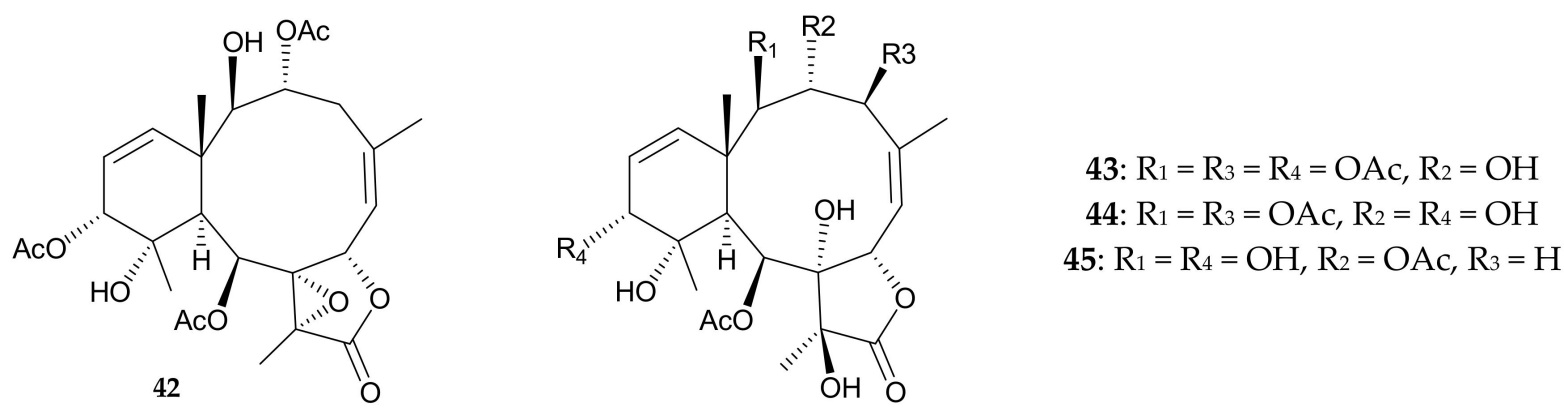
Structures of brianodins A–D (**42**–**45**).

In addition, another group of researchers isolated four new briaranes, brianodins A–D (**42**–**45**), from soft coral identified as *Pachyclavularia* sp. from Okinawa, Japan [20]. They established the structures, including the relative configurations, of briaranes **42**–**45** by interpretation of spectroscopic data, and determined the absolute configurations of brianodins C (**44**) and D (**45**) by the modified Mosher’s method (Figure 10). The 1,2-diol moiety at C-8 and C-17 in **43**–**45** is rarely found in briarane analogues [21,22,23,24,25,26].

**Figure 11:**
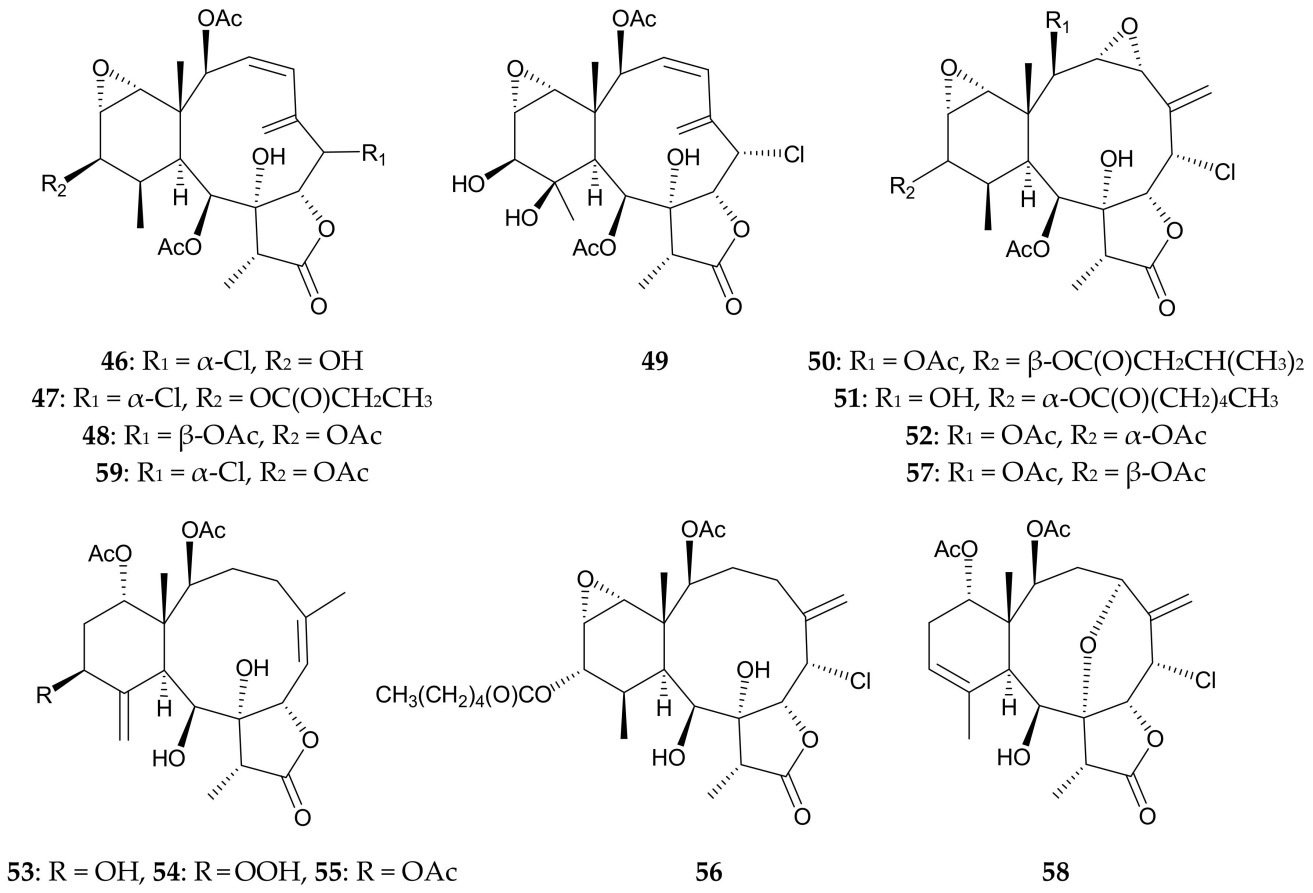
Structures of briaviolides A–J (**46**–**55**), solenolides A (**56**) and D (**57**), excavatolide A (**58**), and briaexcavatolide I (**59**).

Sixteen briarane analogues, including ten new metabolites briaviolides A–J (**46**–**55**), along with six known briaranes, solenolides A (**56**) and D (**57**) [27], excavatolide A (**58**) [28], briaexcavatolide I (**59**) [29] (Figure 11), 4β-acetoxy-9-deacetylstulatulide lactone (**26**) and 9-deacetylstylatulide lactone (**25**) [15] (Figure 7), were isolated from *Briareum violaceum* [2] collected in the waters of Taiwan [30]. Using spectroscopic/chemical methods, the structures, including the relative configurations, of new briaranes **46**–**55** were determined, and the absolute configuration of briaviolide A (**46**) was further confirmed by X-ray crystallographic analysis [30]. In anti-inflammatory activity assays, briaviolides E (**50**) and I (**54**) were found to possess inhibitory effects against the production of superoxide anions by human neutrophils in vitro (inhibition rates = 34.17% and 28.66%, respectively), and inhibited the release of elastase (inhibition rates = 26.03% and 28.81%, respectively) at a concentration of 10 μg/mL; while the monobenzoyl derivative of 46 was found to possess selective activity to inhibit elastase release (inhibition rate = 28.60%) at a concentration of 10 μg/mL [30]. By comparison of the structure-activity relationships between briaranes **53** (briaviolide H) and **54** (briaviolide I), indicating that the hydroperoxy group at C-12 in **54** enhance the activity to inhibit the elastase release and the generation of superoxide anions.

A study performed by Cheng et al. revised the structure of solenolide D (**57**) [31], and this compound was later found to be a potent anti-inflammatory agent with an efficacy comparable to that of indomethacin [27]. Potency in excess of a 70% reduction of edema was observed at a concentration of approximately 15 μg/per ear (mouse ear assay) [27].

## 4. Briarellins from *Pachyclavularia violacea*

**Figure 12:**
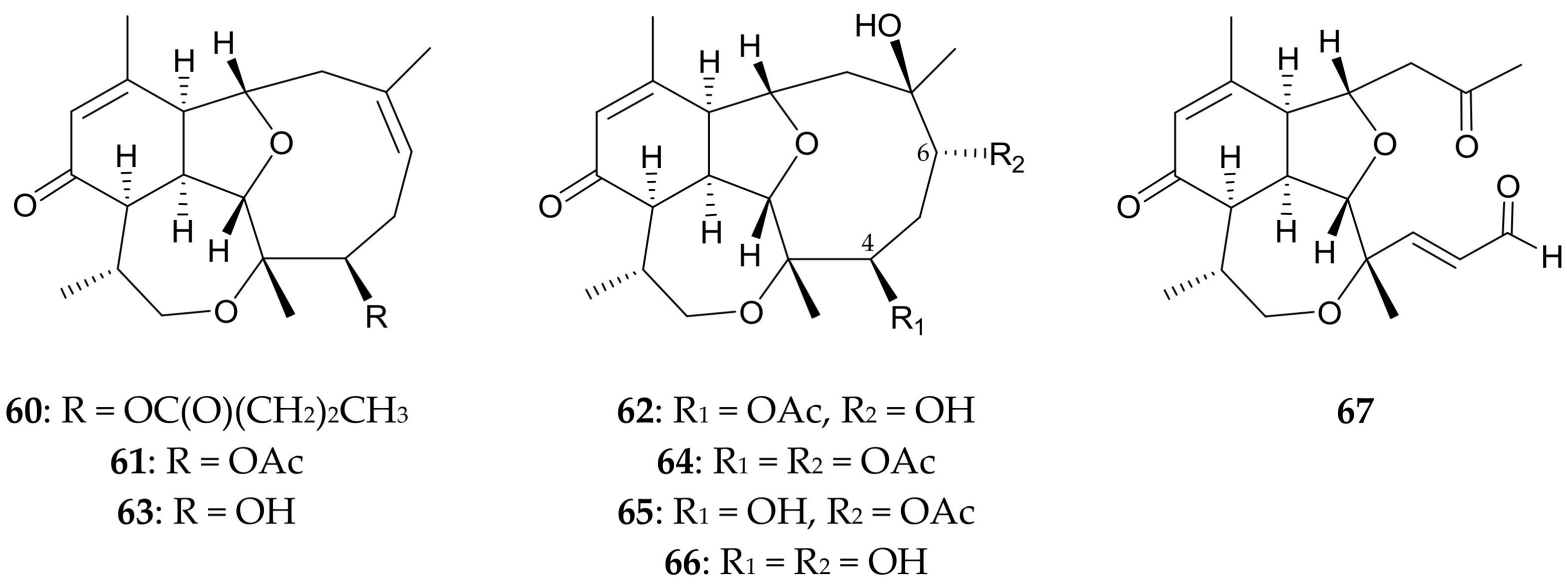
Structures of pachyclavulariaenones A–G (**60**–**66**) and secopachyclavulariaenone A (**67**).

Eight novel briarellins, including pachyclavulariaenones A–G (**60**–**66**) [32,33] and secopachy-clavulariaenone A (**67**) [6] (Figure 12), were isolated from *P. violacea* collected off Kenting Coast, Taiwan. Pachyclavulariaenone B (**61**) was also isolated from *P. violacea* specimens collected in the Togian Islands near Sulawesi Island, Indonesia [34]. The structures, including the relative configurations, of briarellins **60**–**67** were established by spectroscopic methods, and the structures, including the relative configurations, of briarellins **62** (pachyclavulariaenone C) [32] and **65** (pachyclavulariaenone F) [33] were further confirmed by single-crystal X-ray diffraction analyses. Briarellins **60**–**67** were found to be unprecedented examples of this type of compound, as they possess a six-membered carbocyclic ring *cis*-fused to both the ten-membered carbocyclic ring and seven-membered ether ring, making three ring-junction protons *cis* to each other [6,32,33]. Cytotoxic assays revealed that pachyclavulariaenone G (**66**) exhibited cytotoxicity towards P388 and HT-29 tumor cells, with ED_50_ values of 0.2 and 3.2 μg/mL, respectively [33]. It is obvious to note that the hydroxy groups at C-4 and C-6 in 66 caused this compound to be more cytotoxic than its analogues **62**, **64**, and **65**.

## 5. Secosterol from *Pachyclavularia violacea*

**Figure 13:**
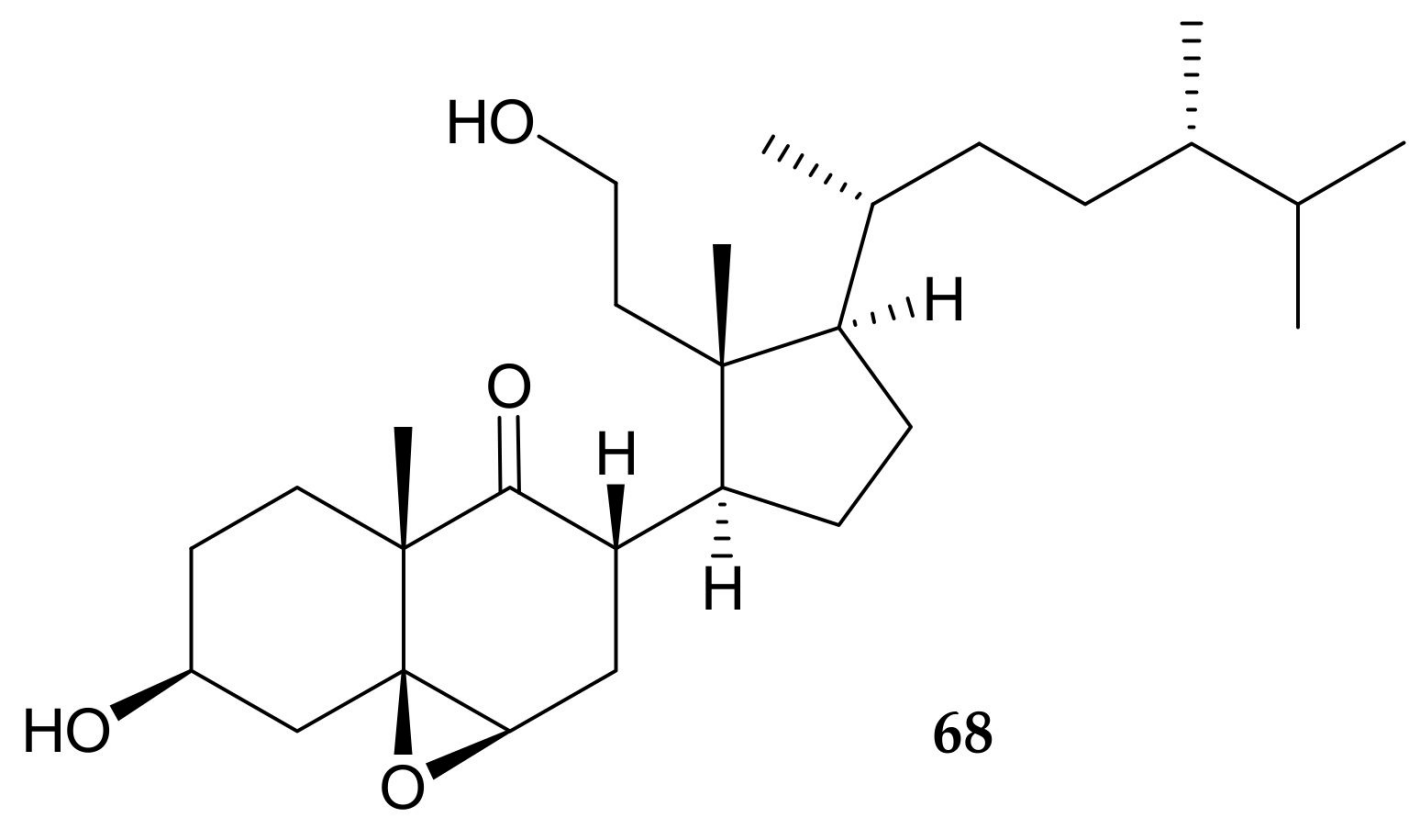
Structure of secosterol **68**.

A new 9,11-secosterol, (24S*)-3β,11-dihydroxy-5β,6β-epoxy-24-methyl-9,11-secocholestan-9-one (**68**) (Figure 13), was isolated from *P. violacea* specimens collected off the Togian Islands, Indonesia, and its structure, including the relative configurations, was elucidated on the basis of spectroscopic data [34].

## 6. Conclusions

Ever since pachyclavulariadiol (**1**) was obtained from a specimen of the octocoral *P. violacea* collected in Australia [4], 68 interesting secondary metabolites, including 20 cembranes (29.41%), 39 briaranes (57.35%), eight briarellins (11.76%), and one secosterol (1.47%), have been isolated from octocorals belonging to the genus *Pachyclavularia*. Many studies have reported that compounds obtained from *Pachyclavularia* spp. possess various biologic activities, such as cytotoxicity (seven cembranes, six briaranes, and one briarellin), anti-inflammatory activity (three briaranes), and ichthyotoxicity (one briarane). It is interesting to note that all compounds from *Pachyclavularia* spp. reported as having been isolated between 1979 and 2014 were collected from octocorals distributed in the Indo-Pacific Ocean. The briarane-type natural products act as a chemical marker of octocorals belonging to the family Briareidae. Up to date, 379 briaranes [35,36,37,38,39,40], 13 eunicellins, 34 asbestinane, one cembrane, and one pyranone [4], have been isolated from the octocorals belonging to the family Briareidae, including the genera *Briareum* and *Erythropodium*. As more than 50% of the compounds obtained from the *Pachyclavularia* genus are briarane-type diterpenoids, we support that the taxonomic position of the *Pachyclavularia* genus should be readjusted and placed under the family Briareidae. Based on above findings, these results suggest that continuing investigation of new terpenoid analogues with the potential bioactivities from this marine organism are worthwhile for further development. The octocoral *Pachyclavularia violacea* had been transplanted to culturing tanks located in the National Museum of Marine Biology and Aquarium, Taiwan, for extraction of additional natural products to establish a stable supply of bioactive material.
